# Severe Type 2 Inflammation Leads to High Platelet-Activating-Factor-Associated Pathology in Chronic Rhinosinusitis with Nasal Polyps—A Hierarchical Cluster Analysis Using Bulk RNA Barcoding and Sequencing

**DOI:** 10.3390/ijms25042113

**Published:** 2024-02-09

**Authors:** Takashi Ishino, Takashi Oda, Tomohiro Kawasumi, Kota Takemoto, Manabu Nishida, Yuichiro Horibe, Nobuyuki Chikuie, Takayuki Taruya, Takao Hamamoto, Tsutomu Ueda, Sachio Takeno

**Affiliations:** Department of Otorhinolaryngology, Head and Neck Surgery, Graduate School of Biomedical Sciences, Hiroshima University, Kasumi 1-2-3, Minami-ku, Hiroshima 734-8551, Japan; tishino@hiroshima-u.ac.jp (T.I.); odataka@hiroshima-u.ac.jp (T.O.); kwtm2022@hiroshima-u.ac.jp (T.K.); kota61@hiroshima-u.ac.jp (K.T.); nm1027@hiroshima-u.ac.jp (M.N.); horibey@hiroshima-u.ac.jp (Y.H.); housejak@hiroshima-u.ac.jp (N.C.); ttaruya@hiroshima-u.ac.jp (T.T.); takao0320@hiroshima-u.ac.jp (T.H.); uedatsu@hiroshima-u.ac.jp (T.U.)

**Keywords:** paranasal sinuses, chronic rhinosinusitis (CRS), BRB-seq, nasal polyps, differentially expressed gene (DEG) analysis, hierarchical analysis, pathway analysis, type 2 inflammation, LPCAT1, LPCAT2, PAFAH1B2, PAFAH1B3, PAFAH2, PTAFR

## Abstract

Platelet-activating factor (PAF) is a phospholipid-derived inflammatory mediator that triggers various inflammatory conditions, including eosinophil activation and recruitment. This study aimed to evaluate the expressions of PAF-metabolism-associated genes, namely genes coding the enzymes involved in PAF synthesis (LPCAT1, LPCAT2, LPCAT3, and LPCAT4), PAF degradation (PAFAH1B2, PAFAH1B3, and PAFAH2), and the gene for the PAF receptor (PTAFR) in subtypes of CRSwNP classified by clinical- or hierarchal-analysis-based classifications. Transcriptomic analysis using bulk RNA barcoding and sequencing (BRB-seq) was performed with CRSwNP, including eosinophilic CRS (ECRS) (*n* = 9), nonECRS *(n* = 8), ECRS with aspirin-exacerbated respiratory disease (Asp) (*n* = 3), and controls with a normal uncinate process mucosa (*n* = 6). PTAFR was only upregulated in ECRS and nonECRS. In the hierarchical cluster analysis with clusters 1 and 2 reflecting patients with low-to-moderate and high levels of type 2 inflammation, respectively, cluster 1 exhibited a significant downregulation of LPCAT2 and an upregulation of PTAFR expression, while cluster 2 showed an upregulation of LPCAT1, PAFAH1B2, and PTAFR and downregulation of PAFAH2 expression. Understanding this strong PAF-associated pathophysiology in the severe type 2 inflammation group could provide valuable insights into the treatment and management of CRSwNP.

## 1. Introduction

Chronic rhinosinusitis (CRS) with nasal polyps (CRSwNP) is a complex inflammatory disorder characterized by diverse clinical presentations and underlying inflammatory profiles. It can be categorized into eosinophilic CRS (ECRS) and non-ECRS based on the extent of eosinophilic infiltration in the sinonasal tissue [1]. Associations between the inflammatory endotypes of types 1, 2, and 3 and the phenotypes of CRSwNP are deeply associated with the pathophysiology of CRSwNP [2]. ECRS, a subtype characterized by Th2-dominant inflammation, often results in less favorable surgical outcomes [1,3], and cases of ECRS with aspirin-exacerbated respiratory disease (AERD) usually exhibit more severe surgical outcomes due to severe type 2 inflammation [4].

The precise mechanism behind the formation and maintenance of nasal polyps in CRSwNP remains unclear, but there is strong evidence suggesting an association with type 2 inflammation. The pathophysiology of ECRS, especially in ECRS with AERD, involves dysregulation in the synthesis of glycerophospholipid mediators, including leukotrienes, prostaglandin, thromboxane, and PAF [5]. In the context of type 2 inflammatory reactions, IgE-mediated hypersensitivity triggers immune cells to process allergens, prompting a B-cell-mediated immune response. This leads to the cross-linking of IgE antibodies on inflammatory cells, initiating mast cell degranulation and the release of various mediators such as PAF, histamine, and pro-inflammatory cytokines [6,7]. These mediators induce both early- and late-phase allergic responses, with the recruitment of eosinophils, basophils, and monocytes resulting in cellular inflammation. PAF, a phospholipid-derived inflammatory mediator released by various cell types [8], plays a pivotal role in multiple inflammatory states, recruiting eosinophils and neutrophils, triggering leukocyte degranulation and adhesion, and generating free radicals such as superoxide and hydroxyl anions in the nasal mucosa [9]. A previous report mentioned that a large amount of PAF was contained in nasal polyps from CRSwNP patients with AERD, and that a high PAF level was correlated with tissue eosinophilia [10]. Moreover, PAF contributes to mast cell activation, which is often linked to severe anaphylactic responses [9]. Its role in inducing vascular permeability leads to nasal hyperreactivity and congestion [11,12,13].

PAF is tightly regulated and produced by two distinct pathways: (1) the de novo pathway, which constitutively produces low levels of PAF, and (2) the remodeling pathway, which rapidly synthesizes the majority of PAF by removing fatty acids (typically arachidonic acid) from the sn-2 position of phosphatidylcholine (PC), a membrane phospholipid. This results in lyso-PAF, followed by the conversion of lyso-PAF into PAF using LPCAT1 and LPCAT2 [14,15]. The reverse reaction from lyso-PAF to PC is catalyzed by LPCAT1–4 acyltransferase activity [16]. PAF is synthesized by a variety of cells, including endothelial cells, platelets, macrophages, monocytes, neutrophils, and eosinophils [17], and demonstrates its biological effects at high picomolar concentrations; the typical serum concentration of PAF in healthy individuals is 400 pg/mL [18].

Produced PAF is rapidly degraded to lyso-PAF by PAF acetylhydrolases (PAFAHs), a family of Ca^2+^-independent lipoprotein-associated A2 phospholipases. The half-life of PAF ranges from 3 to 13 min because PAFAHs regulate PAF activity by hydrolyzing PAF to lyso-PAF as a biologically inactive form [17]. PAFAHs have three isoforms: one is a secreted form (phospholipase A2 Group VII (PLA2G7)), and the other two isoforms are intracellular forms (PAFAH Ib and PAFAH II) [19]. PLA2G7 and PAFAH II can non-specifically hydrolyze oxidatively fragmented phospholipids with potent biological activities, while PAFAH Ib displays high specificity for the sn-2 acetyl group of glycerophosphocholine (GPC), including PAF [20]. On the other hand, there has been no research concerning the association between allergic conditions and PLA2G7 activity, although increased PLA2G7 activity has been reported in various pathologic conditions, including ischemic stroke, myocardial infarction, familial HDL deficiency, chronic cholestasis, diabetes mellitus, rheumatoid arthritis, essential hypertension, and peripheral vascular disease [20]. Therefore, currently, PLA2G7 is thought to be separate from PAF-metabolism-associated genes in allergic conditions.

PAF effects are mediated by binding to the PAF receptor (PTAFR), leading to the mobilization of intracellular calcium ions and the activation of kinases. The activation of PTAFR leads to the activation of cytoplasmic PLA2, with the subsequent formation of leukotrienes, prostaglandins, and thromboxane after the cleavage of arachidonic acid [19]. PTAFR is expressed in T lymphocytes, monocytes, macrophages, platelets, the tracheal epithelium, the vascular endothelium, the lung alveolar wall, the liver, the small intestine, the heart, skeletal and smooth muscle, brain microglia and neurons, the myometrium, the spleen, and the kidney [21,22]. PTAFR expression is regulated by intracellular cyclic AMP, which can downregulate PTAFR gene expression and reduce PAF-induced arachidonic acid release [23,24].

Stimulation of the PAF receptor induces the production of nitric oxide [25], histamine release from basophils, activation and degranulation of mast cells, chemotaxis of mast cells and eosinophils, recruitment of neutrophils, production of IL-4 by B lymphocytes, bronchial smooth muscle contraction, and mucus secretion, as well as increased vascular permeability [19]. The role of PAF in the pathogenesis of allergic rhinitis has been demonstrated [26], but the role of PAF in the pathogenesis of CRSwNP was underestimated in that study. The intricate relationship between PAF and the pathophysiology of CRSwNP, which includes ECRS, with AERD often exhibiting an anaphylactic reaction after the administration of NSAIDS, suggests a potential link between PAF metabolism and its pathophysiology, including the formation and maintenance of nasal polyps derived from vascular permeability associated with plasma extravasation [11,12,13,27].

The current classification methods, such as the JESREC criteria, attempt to stratify ECRS based on clinical findings and eosinophil count [1]. Transcriptome analyses have revealed that the expression levels of type 2 inflammatory cytokines and chemokines do not align with these clinical classifications [28]. These analyses have unveiled significant differences between the clinical classification and the severity of type 2 inflammation. Therefore, this study aimed to evaluate the expressions of genes associated with PAF metabolism, namely those of enzymes involved in PAF synthesis (LPCAT1, LPCAT2, LPCAT3, and LPCAT4) and degradation (PAFAH1B2, PAFAH1B3, and PAFAH2), as well as the gene of the PAF receptor (PTAFR) across subtypes of CRSwNP classified by clinical- or hierarchal-analysis-based classifications. Identifying disparities in PAF-metabolism-associated gene expressions could be crucial for understanding the heterogeneity of CRSwNP, and it may potentially guide more tailored therapeutic interventions.

## 2. Results

### 2.1. Gene Expression Profiles of NPs among ECRS, Asp, and nonECRS

#### Principal Component Analysis (PCA), Heatmap of the Correlation Matrix, and Hierarchical Cluster Analysis

**Figure 1:**
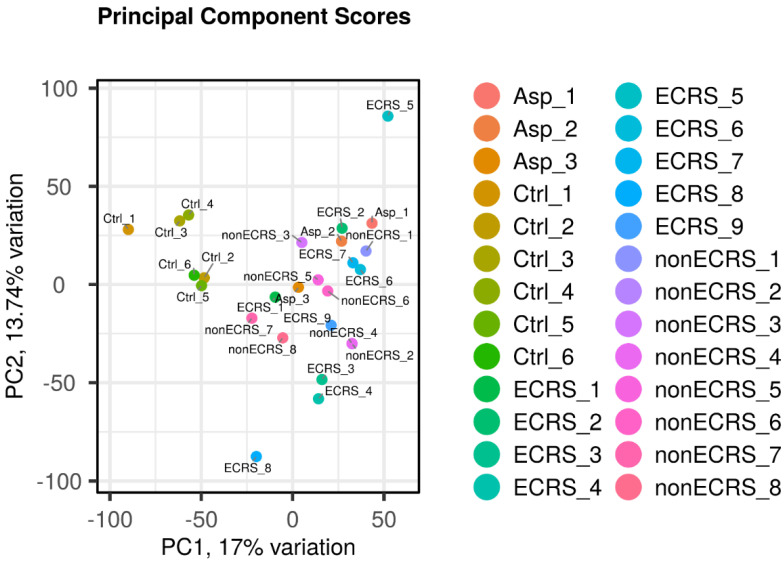
PCA performed among all groups. The control group was clearly segregated, but ECRS, nonECRS, and Asp were not segregated from each other.

PCA was conducted across all groups (Table 1), indicating distinct segregation for the control group, while no significant differences were found among ECRS, Asp, and nonECRS (Figure 1).

These findings support the rationale for the inclusion of ECRS and Asp as part of the same ECRS group in the subsequent analyses of PAF metabolism genes.

### 2.2. Segregation of All CRSwNPs into Two Clusters Using Hierarchical Cluster Analysis

**Figure 2:**
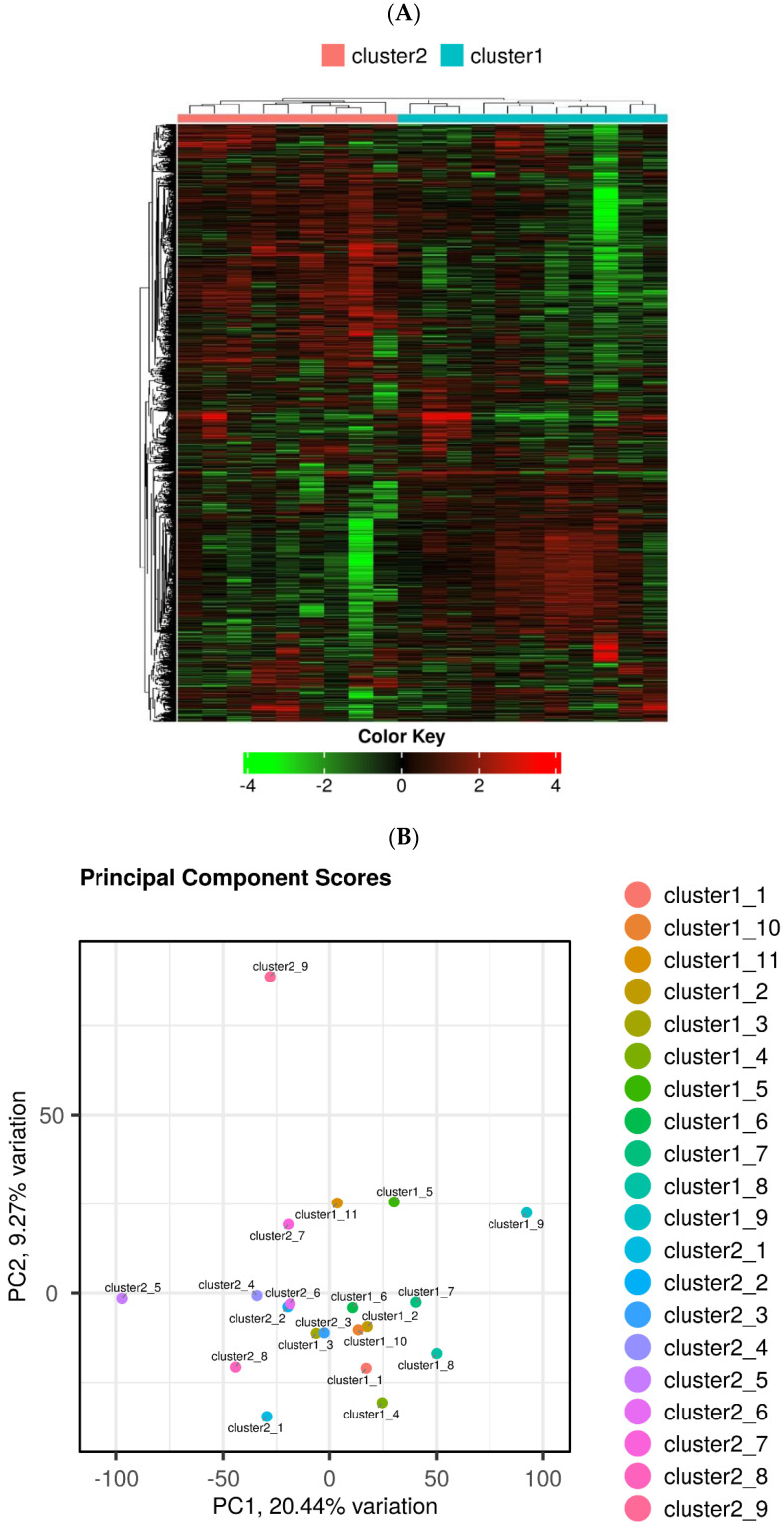
Hierarchic cluster analysis with all CRSwNPs segregated into two clusters (clusters 1 and 2) (**A**). The segregation into two clusters and the comorbidity of asthma were not correlated. The new clusters and control showed clear separation in the PCA analysis (**B**).

**Figure 3:**
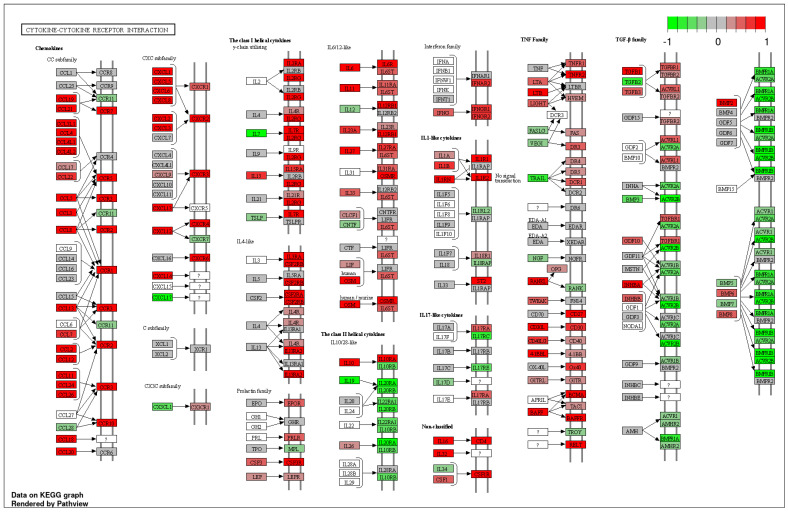
KEGG pathway analysis of cluster 1 vs. cluster 2 in “cytokine-cytokine receptor interaction” by iDEP1.13. Red represents the upregulated DEGs and green represents the downregulated DEGs in cluster 2 compared to cluster 1. The intense result of DEGs including upregulation of interleukin (IL)-4 like cytokine, chemokine (C-C motif) ligand (CCL) 26, and leukotriene B (LTB) suggests that cluster 2 is characterized by severe type 2 inflammation. ? indicates probably unknown.

Hierarchical cluster analysis divided all CRSwNPs into two distinct clusters (referred to as cluster 1 and cluster 2) (Figure 2 and Table 2). However, no correlation was found between the clusters and the presence of asthma as a comorbidity. Notably, these two clusters exhibited significant variations in differentially expressed genes (DEGs) related to a wide array of cytokines and chemokines, indicating the differing severity of type 2 inflammation (Figure 3).

### 2.3. Gene Expression Analysis of PAF-Metabolism-Associated Gene Based on the JESREC-Based Clinical Classification and Clusters Reflecting the Severity of Type 2 Inflammation

**Figure 4:**
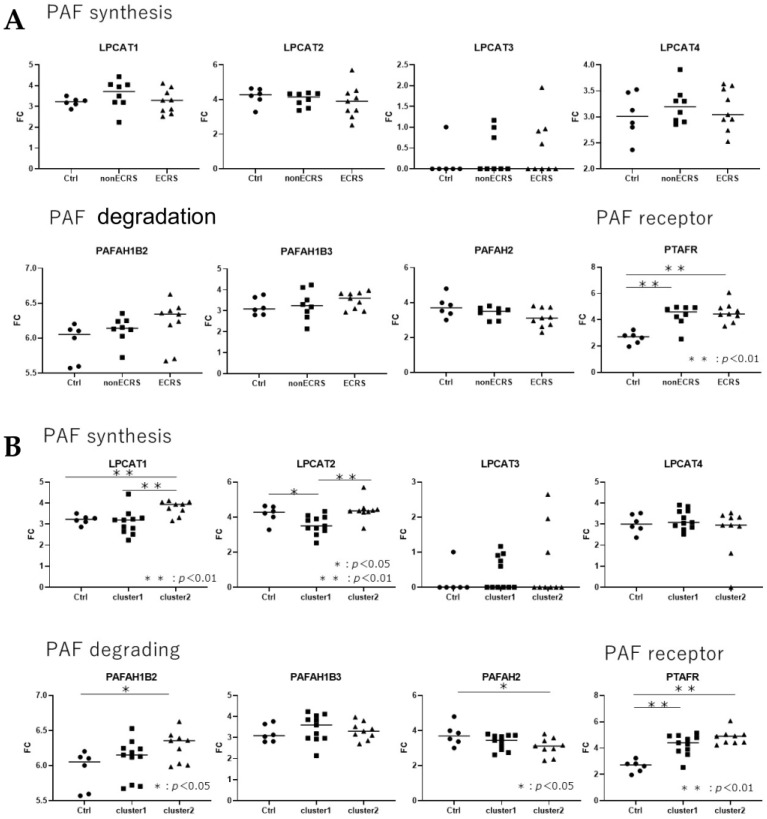
PAF-metabolism-associated gene expression (FC: fold change) in clinical classification with JESREC criteria (**A**) and clusters according to severity of type 2 inflammation (**B**). Clinical classification showed no clear difference among the phenotypes of CRSwNP, but the cluster endotype of CRSwNP demonstrated clear differences in PAF-metabolism-associated gene expression.

When divided into nonECRS and ECRS groups (excluding Asp and Ctrl), the two groups of CRSwNP showed no significant difference in LPCAT1, LPCAT2, LPCAT3, LPCAT4, PAFAH1B2, PAFAH1B3, and PAFAH2, but both groups of CRSwNP showed an upregulation of PTAFR compared to the Ctrl (Figure 4A). The same analysis considering ECRS and Asp as part of the same ECRS group also exhibited the same statistical results. The two clusters reflecting the severity of type 2 inflammation (i.e., clusters 1 and 2) showed no significant difference in LPCAT3, LPCAT4, or PAFAH1B3. However, while cluster 1 exhibited upregulated PTAFR and downregulated LPCAT2 compared to the Ctrl, cluster 2 exhibited upregulated LPCAT1, PAFAH1B2, and PTAFR, and downregulated PAFAH2. Comparing the clusters, cluster 2 showed an upregulation in LPCAT1 and LPCAT2 (Figure 4B).

### 2.4. Analysis between PAF-Metabolism-Associated Gene Expression and Clinical Features

**Figure 5:**
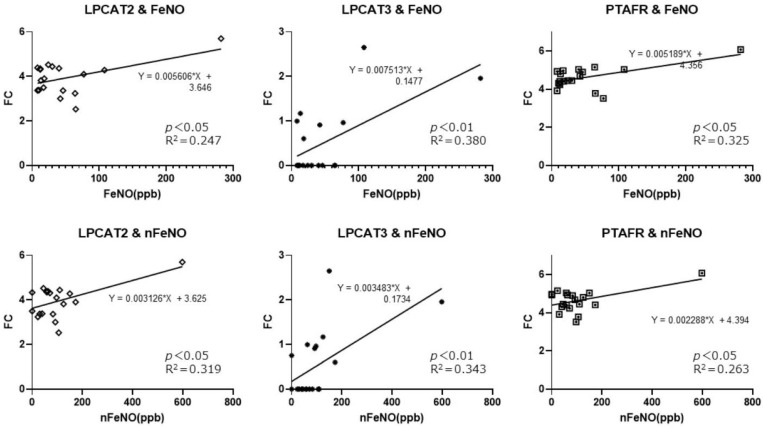
PAF-metabolism-associated gene expression (FC: fold change) in CRSwNPs based on the test of FeNO and nFeNO. FeNO and total nFeNO correlated with LPCAT2, LPCAT3, and PTAFR expressions using the Pearson correlation coefficient, but all data demonstrated low R^2^ in linear regression analysis. FeNO, oral fractional exhaled nitric oxide; nFeNO, total nasal FeNO.

Significant correlations only between oral fractional exhaled nitric oxide (FeNO) or total nasal FeNO (nFeNO) and LPCAT2, LPCAT3, and PTAFR were recognized in CRSwNP (Figure 5), but no significant correlations were observed between either FeNO value and LPCAT1, LPCAT4, PAFAH1B2, or PAFAH1B3 expression. There was also no correlation between tissue eosinophils, blood eosinophils, total IgE, or the JESREC score and expressions of LPCAT1, LPCAT2, LPCAT3, LPCAT4, PAFAH1B2, PAFAH1B3, PAFAH2, or PTAFR genes.

## 3. Discussion

PAF-metabolism-associated gene expression using transcriptome analysis revealed that clinical classification based on the JESREC study exhibited a significant upregulation of PTAFR gene expression in nonECRS and ECRS compared to the Ctrl; however, the other genes showed no differences among nonECRS, ECRS, and Ctrl. Additionally, there was no difference between nonECRS and ECRS in PTAFR gene expression. These results demonstrating significant upregulations in nonECRS and ECRS compared to the Ctrl and no difference between nonECRS and ECRS in PTAFR gene expression have been previously demonstrated [27] and suggest that clinical classification based on the JESREC criteria or status with/without comorbid asthma and/or AERD may be unrelated to PAF metabolism. On the other hand, the upregulation of PTAFR gene expression in nasal polyps from both nonECRS and ECRS suggests that PTAFR upregulation, enhancing the possibility of *PAF*-signaling upregulation, is associated with the formation and/or maintenance of nasal polyps.

However, two clusters from the hierarchical analysis were clearly segregated from each other in the PCA analysis, and they demonstrated a clear difference in a variety of cytokine expressions in the Kyoto Encyclopedia of Genes and Genomes (KEGG) pathway analysis that appeared to reflect the severity of type 2 inflammation. In the cluster analysis of PAF-associated gene expression, cluster 2 exhibited a significant upregulation of LPCAT1, PAFAHB2, and PTAFR and a significant downregulation of PAFAH2 compared to the Ctrl. In contrast, cluster 1 exhibited a significant upregulation of PTAFR and a significant downregulation of LPCAT2. Comparing clusters 1 and 2, cluster 2 showed a significant upregulation of LPCAT1 and LPCAT2 expression.

LPCAT1 and LPCAT2 are the main enzymes for PAF synthesis from lyso-PAF [14,15], and PAFAH2 is the main degrading enzyme, hydrolyzing PAF to the biologically inactive form, lyso-PAF [19]. Although PAFAH1B2 and PAFAH1B3 are capable of hydrolyzing PAF, previous reports have demonstrated that the RNAi-mediated knockdown of PAFAH1B2 or PAFAH1B3 does not alter PAF levels or PAF hydrolytic activity, indicating that these enzymes may possess alternate endogenous substrates [29]. These results suggest that cluster 2, which seems to reflect more severe type 2 inflammation, has a high PAF-associated pathophysiology with the upregulation of PAF synthesis and the downregulation of PAF degradation, leading to local PAF accumulation as well as intensification of the effects of PAF signaling via the upregulation of PTAFR.

**Figure 6:**
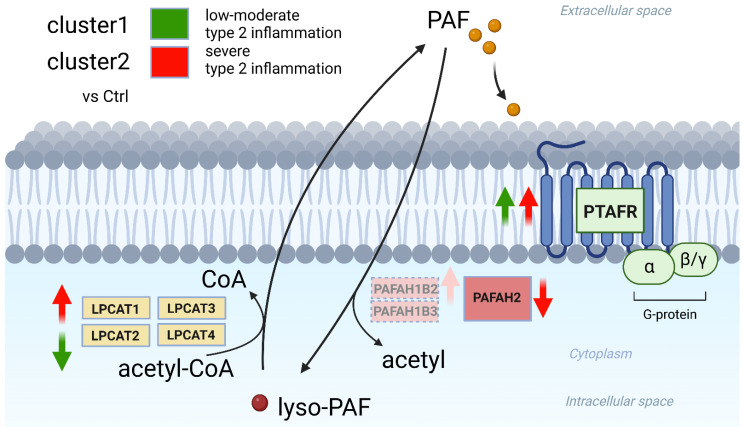
Summary of PAF-metabolism-associated gene expression in CRSwNP. Cluster 2 of CRSwNP, which exhibits severe type 2 inflammation, has a high PAF-associated pathophysiology with the upregulation of PAF synthesis (LPCAT1) and the downregulation of PAF degradation (PAFAH2), leading to local PAF accumulation and intensification of the effects of PAF signaling via the upregulation of PTAFR.

The results showed for the first time that severe type 2 inflammation induced high levels of PAF-associated pathophysiology and that the main cause of the high PAF-associated pathophysiology appears to derive from high lyso-PAF and PAF synthesis via overexpressed LPCAT1 and LPCAT2 compared to the cluster of moderate-to-low type 2 inflammatory types of CRSwNP (cluster 1) (Figure 6).

On the other hand, our results did not reveal any correlation between gene expressions associated with PAF metabolism and blood/tissue eosinophil count or the clinical classification. Only the oral FeNO and total nasal FeNO demonstrated a relationship between LPCAT2, LPCAT3, and PTAFR, but they did not exhibit the above differences in PAF-metabolism-associated gene expression, especially in LPCAT1 and LPCAT2, shown in the cluster analysis. The difference may come from the discrepancy between the severity of type 2 inflammation and blood/tissue eosinophilia, both at the FeNO level, or clinical classification, because the blood/tissue eosinophil count is used as a biomarker of eosinophilia [30], and because FeNO is used for airway inflammation or irritation [31], and these clinical classifications are used for the phenotype diagnosis [1]. Although FeNO and nFeNO reflect the severity of allergic inflammation [31], our results revealed that both FeNOs can reflect the severity of allergic inflammation but not the severity of type 2 inflammation. Direct biomarkers for the severity of type 2 inflammation, which are currently underdeveloped, should be developed to further analyze the pathophysiology in CRSwNP.

Previous reports have mentioned differences in lyso-PAF concentration between normal nasal polyps and nasal polyps with AERD, suggesting an association between airway inflammation and/or disease severity and lyso-PAF concentration [27]. Other papers have also mentioned that human nasal polyps with severe eosinophil infiltration exhibit a high PAF concentration [10]. Our results clearly demonstrated that the group of nasal polyps with severe type 2 inflammation had an increased expression of genes related to PAF metabolism, suggesting that PAF plays an important role in the pathogenesis of CRSwNP by inducing increased tissue neutrophilia and eosinophilia in the nasal mucosa [19]. Therefore, these results support the idea that PAF-metabolism-associated gene expression, especially in combination with LPCAT1 and LPCAT2 gene expression, can become a biomarker for the severity of type 2 inflammation in CRSwNP and may add other insights for strategies of biologic therapy initiation and selection in CRSwNP [32].

There are some limitations to our study. First, due to the sample size, our results may affect the power of DEG detection between ECRS and nonECRS. Second, this study only included Japanese patients; therefore, caution should be taken when extrapolating our results to other ethnic groups. Third, this study did not confirm protein levels, only gene expressions. Additionally, this study did not measure the gene expression level using a digital PCR system, which can avoid erroneous results derived from fluctuations in reference gene expression in real-time PCR. Further studies are needed to analyze the correlation among the CRS phenotype, protein levels, and transcriptional overexpression of PTAFR and other PAF-associated genes.

In summary, this study’s examination of PTAFR gene expression in nasal polyps reveals that severe type 2 inflammation leads to a particularly high PAF metabolism in nasal polyps, which may induce the proliferation and maintenance of nasal polyps. However, due to the use of hierarchical analysis classification, the small sample size, and the limitation of these findings to Japanese patients, further research is needed to confirm our findings and the association between type 2 inflammation and the production of PAF-metabolism-associated proteins, including PAF and/or lyso-PAF, to continue the development of anti-PAF therapies for CRSwNP, especially those with severe type 2 inflammation. Additionally, research concerning LPCAT1 and LPCAT2 gene expression in CRSwNP should be performed to confirm whether these gene expressions are indeed good biomarkers for the severity of type 2 inflammation.

## 4. Materials and Methods

### 4.1. Patient Recruitment

Patients with or without CRS who underwent endoscopic sinus surgery at Hiroshima Medical University Hospital between October 2016 and October 2021 were enrolled. The diagnosis of CRS was based on computed tomography (CT) scanning, patient history, clinical symptoms, and endoscopic findings. The inclusion criteria for CRSwNP were as follows: no use of oral/nasal steroids within 4 weeks before surgery; no improvement in continuous nasal drip; and post-nasal drip and nasal congestion after medical treatment, including low-dose macrolide therapy. Patients with CRSwNP were clinically diagnosed as ECRS or nonECRS based on the diagnostic criteria of the JESREC study [1]. The diagnostic criteria for ECRS (JESREC score) included (1) side affected: both sides, 3 points; (2) with nasal polyps, 2 points; (3) CT changes: ethmoid/maxillary ≥ 1, 2 points; and (4) peripheral blood eosinophil count (%): 2< and ≤5%, 4 points; 5< and ≤10%, 8 points; >10%, 10 points. Eosinophil infiltration into the NPs was diagnosed by calculating the mean cell count of the 3 densest areas of eosinophils in the hematoxylin and eosin (HE)-stained sections under ×400 magnification (ocular lens, field number 22). The diagnosis of ECRS was made based on both the total JESREC score of 11 points or higher and the eosinophil infiltration count into the NPs of 70 or more [1]. The group of ECRS with aspirin-exacerbated respiratory disease was independently classified as Asp (N = 3), and the others were classified as ECRS (*n*= 9) and nonECRS (*n* = 8). The control patients (Ctrl) (*n*= 6) were also diagnosed based on their anatomical abnormalities, but they showed no inflammatory mucosal change or bacterial infection of the uncinate process. The exclusion criteria for CRSwNP were as follows: treatment with oral/nasal steroids within 4 weeks before surgery, fungal/allergic fungal rhinosinusitis, and primary ciliary dyskinesia. Medical data on clinical information, demographics, comorbid conditions, and results of laboratory tests were collected from the medical records. Smoking history was defined as a positive response for current and/or previous habit of smoking, and comorbid asthma was defined as positive for either aspirin-tolerant or -intolerant asthma. FeNO levels were analyzed using a NIOX VERO^®^ (Aerocrine AB, Solna, Sweden) following the recommendations of the European Respiratory Society/American Thoracic Society, and measurement of nFeNO was conducted as previously described [33].

### 4.2. RNA-Seq Using BRB-Seq

BRB-seq [34] was performed for library preparation with the following modifications: Barcoded oligo-dT-based primer (5′-GCCGGTAATACGACTCACTATAGGGAGTTCTACAGTCCGACGATCNNNNNNNNNNCCCCCCCCCTTTTTTTTTTTTTTTTTTTTTTTTV-3′; 10 bp “N” = UMI, 9 bp “C” = cell barcode) was used for single-stranded synthesis. A second-strand synthesis module (NEB, #E6111) was used for double-stranded cDNA synthesis. In-house MEDS-B Tn5 transposase [27,28] was used for augmentation, and libraries were amplified for 10 cycles of PCR using Phusion High-Fidelity DNA Polymerase (Thermo Scientific, Waltham, MA, USA, #M0530) with the following primers (5′-AATGATACGGCGACCACCGAGATCTACACindexGTTCAGAGTTCTACAGTCCGA-3′, 5′-CAAGCAGAAGACGGCATACGAGATindexGTCTCGTGGGCTCGGAGATGT-3’). An Illumina NovaSeq6000 was used to obtain 15 bp of barcode read (Read1) and 81 bp of insert read (Read2).

### 4.3. Data Processing of BRB-Seq

Read1 (barcode read) was extracted using UMI-tools (ver.1.1.1) with the command “umi_tools extract -I read1.fastq --read2-in=read2.fastq --bc-pattern=NNNNNNNNNNCCCCCCCCC –read2-stdout”. Adaptor sequences and low-quality sequences were removed and read lengths below 20 bp were discarded using Trim Galore (ver.0.6.7). Reads were mapped to the GRCh38 reference using HISAT2 (ver.2.2.1). Read counts for each gene were obtained by featureCounts (ver. 2.0.1), and UMI duplication was removed by UMI-tools with the command “umi_tools count –method=unique --per-gene --per-cell--gene-tag=XT”. The normalized read count value was obtained by using DESeq2 (ver. 1.34.0). Gene-level expression data (read counts) were processed using the web portal for integrated differential expression and pathway analysis (iDEP.1.13; http://bioinformatics.sdstate.edu/idep11 (accessed on 31 December 2023). iDEP.1.13 was used for Principal Component Analysis (PCA), hierarchical clustering with a heatmap by center genes, identification of differentially expressed genes (DEGs), and pathway analysis. DEGs were extracted with an FDR cutoff of 0.1 and min-fold change of 2. Pathway analysis was performed using GAGE with hsa04060 “cytokine–cytokine receptor interaction” of the Kyoto Encyclopedia of Genes and Genomes (KEGGs) database. Geneset size was set to a minimum of 5 and a maximum of 2000, and the pathway significance cutoff (FDR) was set to 0.2.

### 4.4. Data Analysis for PAF-Metabolism-Associated Gene Expression

In the category of fold change in CRSwNP, expressions of genes associated with PAF synthesis (LPCAT1, LPCAT2, LPCAT3, and LPCAT4) and degradation (PAFAH1B2, PAFAH1B3, and PAFAH2), along with the gene for the PAF receptor (PTAFR), were measured and analyzed in relation to clinical classification and hierarchical classification. Statistical analysis was performed using the chi-squared distribution, Welch’s *t* test, Pearson correlation coefficient, and linear regression analysis with Graphpad prism 8.4.3.

## 5. Conclusions

This study shed light on PAF metabolism as a marker of the severity of type 2 inflammation. Additionally, this study revealed that hierarchical-analysis-based classification as an endotype categorization was useful to elucidate the differences in CRSwNP. Our results should provide valuable data for future studies elucidating other differences in the endotype of CRSwNPs, future therapies for CRSwNP using anti-PAF metabolism drugs, and the potential use of LPCAT1 and LPCAT2 as biomarkers for the severity of type 2 inflammation.

## Figures and Tables

**Table 1:** Patient demographics. BMI, body mass index; NP, nasal polyp. FeNO, oral fractional exhaled nitric oxide; nFeNO, total nasal FeNO.

Patients	Control_1	Control_2	Control_3	Control_4	Control_5	Control_6	nonECRS_1	nonECRS_2	nonECRS_3	nonECRS_4	nonECRS_5	nonECRS_6	nonECRS_7	nonECRS_8
cluster							2	1	2	1	1	2	1	1
height (cm)	153.4	149	158	151.6	145	151.7	165	175.2	162.4	156.8	172	159.5	166.6	169
weight (kg)	52.3	46.6	58.8	53	58.8	41.6	64.2	77.8	49.4	54.4	69.5	61.7	67.9	61
BMI	22.2	21.0	23.6	23.1	28.0	18.1	23.6	25.3	18.7	22.1	23.5	24.3	24.5	21.4
sex	F	F	F	F	F	F	M	M	F	F	M	M	M	F
age	64	66	39	70	61	69	53	44	66	67	47	54	69	40
comorbid asthma (1: positive)	0	0	0	0	0	0	0	0	0	0	0	0	0	0
olfactory disturbance (1: positive)	0	0	0	0	0	0	0	0	0	1	0	0	0	1
smoking history (1: positive)	0	0	0	0	0	0	0	1	0	0	0	1	0	0
blood eosinophil (%)	0.9	1.5	0.6	0.2	1.6	1.2	1.3	2.9	0.3	0.3	1.6	1.6	3.6	1.6
total IgE (IU/mL)	467	85.6	NA	142	NA	NA	477	73.2	22	54.8	14.9	860	506	15.6
total NP score							0	5	2	8	3	0	2	5
total CT score							11	10	4	24	12	6	16	17
tissue eosinophils of nasal polyp (cell count/HPF)							12.0	6.6	15.0	38.0	70.3	0.0	NA	6.0
JESREC score							3	9	0	5	7	0	9	5
FeNO (ppb)							8	13	12	17	NA	12	27	8
nFeNO (ppb)							63	125	71	0	0	59	57	30
**Patients**			**ECRS_1**	**ECRS_2**	**ECRS_3**	**ECRS_4**	**ECRS_5**	**ECRS_6**	**ECRS_7**	**ECRS_8**	**ECRS_9**	**Asp_1**	**Asp_2**	**Asp_3**
cluster			1	2	1	1	2	2	2	1	1	2	2	1
height (cm)			173.7	154.2	166.9	146.7	172.2	168	145.5	162.1	162	162	156.5	150
weight (kg)			80.5	55.9	57.3	40.1	57.9	62.7	68.3	44	64.5	50	46.3	49.7
BMI			26.7	23.5	20.6	18.6	19.5	22.2	32.3	16.8	24.6	19.1	18.9	22.1
sex			M	F	M	M	F	M	F	F	M	F	F	F
age			43	52	51	63	31	48	61	47	70	60	42	67
comorbid asthma (1: positive)			1	1	1	0	1	1	1	1	1	1	1	1
olfactory disturbance (1: positive)			1	1	1	1	0	0	1	1	1	1	1	1
smoking history (1: positive)			1	0	1	1	0	0	0	0	0	0	0	0
blood eosinophil (%)			11.6	10.2	5.5	1.9	9.6	19.5	1.1	6.1	0.6	20	11.2	10.1
total IgE (IU/mL)			319	208	444	536	24.3	66.7	122	72.6	255	71.1	462	212
total NP score			4	2	6	8	6	6	6	6	2	7	6	6
total CT score			16	10	10	16	9	24	22	24	21	19	27	26
tissue eosinophils of nasal polyp (cell count/HPF)			76.0	73.0	110.0	83.0	58.3	220.0	117.0	145.0	140.0	416.0	170.0	170.0
JESREC score			17	17	15	11	15	15	11	15	17	17	17	17
FeNO (ppb)			77	24	65	10	282	46	40	42	18	30	108	64
nFeNO (ppb)			97	45	106	40	598	83	58	92	173	110	150	23

**Table 2:** Patient demographics of clusters 1 and 2 (mean ± SD). BMI, body mass index; NP, nasal polyp; FeNO, oral fractional exhaled nitric oxide; nFeNO, total nasal FeNO. Statistical analysis of sex, comorbid asthma, olfactory disturbance, and smoking history was performed with chi-squared distribution, and other factors were analyzed using Welch’s *t* test.

	Cluster 1	Cluster 2	*p* Value
n	11	9	
height (cm)	163.727 ± 9.3681	160.589 ± 7.9126	0.435
weight (kg)	60.609 ± 13.0525	57.378 ± 7.5506	0.52
BMI	22.372 ± 2.9651	22.448 ± 4.3077	0.963
sex (men/women)	7/4	3/6	0.37
age	55.273 ± 11.8583	51.889 ± 10.3236	0.515
comorbid asthma	positive: 5	positive: 6	0.406
olfactory disturbance	positive: 8	positive: 4	0.362
smoking history	positive: 4	positive: 1	0.319
blood eosinophil (%)	4.164 ± 3.7932	8.311 ± 7.7924	0.136
total IgE (IU/mL)	227.555 ± 199.1742	257.011 ± 286.0960	0.789
total NP score	5.0 ± 2.0976	3.889 ± 2.8480	0.328
total CT score	17.455 ± 5.6456	14.667 ± 8.4261	0.388
tissue eosinophil of nasal polyp (cell count/HPF)	84.49 ± 57.126	120.14 ± 133.805	0.452
JESREC score	11.545 ± 4.8242	10.556 ± 7.4517	0.724
FeNO (ppb)	34.10 ± 25.951	62.44 ± 87.777	0.342
nFeNO (ppb)	67.545 ± 55.4596	137.444 ± 175.6966	0.227

## Data Availability

Data is contained within the article.

## Abbreviations

AERD, aspirin-exacerbated respiratory disease; BRB-seq, bulk RNA; barcoding and sequencing; CCL, chemokine (C-C motif) ligand; CRSwNP, chronic rhinosinusitis with nasal polyps; DEG, differentially expressed gene; ECRS, eosinophilic CRS; FC, fold change; FeNO, fractional exhaled nitric oxide; IL, interleukin; JESREC, Japanese Epidemiological Survey of Refractory Eosinophilic Chronic Rhinosinusitis; KEGG, Kyoto Encyclopedia of Genes and Genomes; LPCAT, lysophosphatidylcholine acyltransferase; LTB, leukotriene B; nFeNO, total nasal FeNO; PAF, platelet-activating factor; PAFAH, PAF acetylhydrolases; PCA, Principal Component Analysis; PLA2G7, phospholipase A2 Group VII; PTAFR, platelet-activating factor receptor.
